# Gender Dictates the Relationship between Serum Lipids and Leukocyte Counts in the National Health and Nutrition Examination Survey 1999–2004

**DOI:** 10.3390/jcm8030365

**Published:** 2019-03-15

**Authors:** Catherine J. Andersen, Terrence M. Vance

**Affiliations:** 1 Department of Biology, Fairfield University, Fairfield, CT 06824, USA; 2 Department of Nutrition and Dietetics, The State University of New York Plattsburgh, Plattsburgh, NY 12901, USA; tvanc001@plattsburgh.edu

**Keywords:** serum lipids, cholesterol, leukocytes, gender, NHANES

## Abstract

Dyslipidemias and leukocytosis are associated with cardiovascular disease and immune disorders. Mechanistic studies have shown lipoprotein metabolism to play a significant role in the regulation of atherosclerosis development and leukocyte activation, whereas lipid-lowering treatments have been shown to exert beneficial anti-inflammatory and immunomodulatory effects in clinical trials. However, the relationship between clinical markers of lipid metabolism and leukocyte counts has not been extensively evaluated at the population level. We aimed to determine whether clinical blood lipid measures are associated with leukocyte counts in the general U.S. population represented in the National Health and Nutrition Examination Survey (NHANES) 1999–2004, and whether differences exist between men and women (*n* = 5647). We observed a strong positive linear trend between serum triglycerides vs. blood lymphocyte and basophil counts in both men and women, whereas a positive trend between monocytes vs. triglycerides and lymphocytes vs. total cholesterol and LDL-cholesterol (LDL-C) was only detected in women. Conversely, HDL-C was inversely associated with a greater number of leukocyte subsets in men, whereas inverse trends between HDL-C vs. lymphocytes were observed in both men and women. In multiple regression models, a 10% increase in total cholesterol, LDL-C, and triglycerides was associated with a predicted 1.6%, 0.6%, and 1.4% increase in blood lymphocyte counts in women, respectively, whereas no relationship was observed in men. In both men and women, a 10% increase in triglycerides was additionally associated with higher lymphocyte, neutrophil, and basophil counts, whereas 10% increases in HDL-cholesterol were associated with significantly lower lymphocyte, neutrophil, eosinophil, and basophil counts in men, in addition to lower lymphocyte and monocyte counts in women. These findings suggest that clinical lipid markers may be used to predict blood leukocyte distributions, and that a gender-specific relationship exists between distinct classes of serum lipids and immune cell subsets.

## 1. Introduction

Leukocytosis, or elevation of leukocyte counts, serves as a marker of immune activation and inflammation [1]. While commonly observed in infection [2], injury [3], hematological malignancies [4], and allergy [5], elevated peripheral blood leukocyte counts have additionally been associated with increased risk of cardiovascular disease (CVD) [6,7,8], type 2 diabetes mellitus [9], metabolic syndrome [10,11,12], and nonalcoholic fatty liver disease [13,14]. Altered leukocyte profiles not only contribute to metabolic dysfunction and inflammation, but may impair immune responses to acute infection and injury [15,16]. Thus, it is essential to understand the complexity of factors that impact leukocyte activation, expansion, and inflammation in order to develop effective strategies to mitigate metabolic and immune disorders [17].

Evidence from clinical trials and mechanistic studies suggests that there is a direct relationship between lipid metabolism and leukocyte profiles, and that lipid-lowering interventions may provide therapeutic potential to markers of leukocytosis and autoimmunity [18,19]. Lipoproteins can modulate the activity of circulating blood leukocytes through interactions with immunomodulatory proteins [20,21,22,23], as well as through mediation of cellular lipid efflux and/or loading via mechanisms that have been elucidated within the context of lipid-laden arterial macrophages in atherosclerosis. Greater cholesterol loading of leukocytes has been shown to enhance lipid raft formation, cellular proliferation, and inflammatory potential, whereas promotion of cholesterol depletion, either through HDL-mediated efflux or statin-induced suppression of endogenous cholesterol synthesis, has been shown to suppress proliferation and pro-inflammatory immune responses [24,25,26,27,28]. Thus, dyslipidemias may directly exasperate leukocytosis and altered leukocyte profiles associated with chronic metabolic disease and immune disorders [23,29,30,31].

Despite promising findings from clinical and mechanistic studies, along with population studies from China [32], Taiwan [33], and Japan [34], the relationship between serum cholesterol markers and leukocyte profiles has not been extensively evaluated in the general U.S. adult population, or in comparison between genders. Given that serum lipid profiles [35,36], CVD risk [37], autoimmune disease incidence [38], and immune responses to infectious pathogens and viral vaccines vary between men and women [39,40], research is warranted to determine whether the relationship between clinical lipid and immune biomarkers is gender-specific to help inform diagnostic and therapeutic strategies. Thus, our objective was to investigate the association between serum lipids and clinical leukocyte counts in men and women from the National Health and Nutrition Examination Survey (NHANES) 1999–2004. We hypothesized that blood lipid markers can be used to predict leukocyte profiles, and that variations in the lipid-immune relationship would be observed between men and women.

## 2. Experimental Section

### 2.1. Study Design and Population

NHANES is a comprehensive, cross-sectional, annual survey conducted in the U.S. civilian, noninstitutionalized population by the Center for Disease Control National Center for Health Sciences. Data from men and women ≥20 years old (*n* = 5647) who participated in NHANES 1999–2000, 2001–2002, and 2003–2004 were used for post-hoc analyses. Detailed information, protocols and datasets are available online at: https://www.cdc.gov/nchs/index.htm. All NHANES protocols were approved by the National Center for Health Statistics Research Ethics Review Board, and each participant provided informed consent.

### 2.2. Survey Data and Sample Collection

Data on participant education level, race/ethnicity, statin use, and age, was collected via survey by trained interviewers during NHANES 1999–2004 data collection cycles. Fasting blood samples were collected in mobile medical units, and blood samples were collected for measurement of fasting serum lipids, differential blood cell counts, and serum cotinine—a marker of tobacco smoking [41]. Serum cotinine (ng/mL) was by determined by isotope dilution–high performance liquid chromatography/atmospheric pressure chemical ionization tandem mass spectrometry (ID HPLC–APCI MS/MS). Waist circumference was measured using a non-flexible tape, and body weight and height were determined to calculate body mass index (BMI).

### 2.3. Fasting Serum Lipids

As stated in NHANES protocols specific to each survey cycle, total cholesterol and triglycerides were measured directly from fasted serum samples using enzymatic assays. Serum HDL-cholesterol (HDL-C) was measured using enzymatic assays following apolipoprotein B-containing lipoprotein depletion by heparin-Mn^2+^ precipitation or direct HDL immunoassay. Total cholesterol, HDL-C, and triglyceride concentrations were expressed as mg/dL. LDL-cholesterol (LDL-C) was calculated using the Friedewald equation: (LDL-C) = ((total cholesterol) − (HDL-C) − (triglycerides))/5 and expressed as mg/dL [42]. Clinically-relevant lipid categories were as follows: total cholesterol (Optimal: <200 mg/dL; Borderline High: 200 to <240 mg/dL; High: >240 mg/dL), LDL-C (Optimal: <100 mg/dL; Near/Above Optimal: 100 to <130 mg/dL; 130 to <160 mg/dL, 160 to <190 mg/dL, >190 mg/dL), HDL-C (High/Optimal: Men: ≥40 mg/dL, Women: ≥50 mg/dL; Low: Men: <40 mg/dL, Women: <50 mg/dL), and triglycerides (<150 mg/dL; 150 to <200 mg/dL; ≥200 mg/dL) [35,43].

### 2.4. Differential Leukocyte Counts

Complete blood counts with 5-part differential measures were performed on whole blood samples collected during NHANES 1999–2004 cycles using a Beckman Coulter MAXM instrument. The 5-part differential measure provided cell numbers of lymphocytes, monocytes, segmented neutrophils, eosinophils, and basophils (10^3^ cells/μL) that were used in post-hoc analyses.

### 2.5. Statistical Analysis

All statistical analyses were performed using SAS version 9.4 (SAS Institute Inc., Cary, NY, USA). SAS SURVEY procedures were used to account for the complex probability sample of NHANES. Analyses were restricted to subjects for which data was available on all outcomes and predictors. Missing values were treated as not missing completely at random. Because of the influence of sex on serum lipids, all analyses were performed separately for men and women. Descriptive statistics were calculated separately for men and women in the sample and reported as counts and percentages for categorical variables and medians and interquartile ranges for continuous variables. Comparison of descriptive statistics between men vs. women was performed by Chi-square tests for categorical variables, and *t*-tests for continuous variables. Analysis of covariance (ANCOVA) was used to determine associations between peripheral blood leukocytes and categories of serum lipids, with blood leukocyte levels log transformed (base e) to achieve a normal distribution of residuals. Multiple linear regression was used to determine associations between peripheral blood leukocytes and serum lipids on a continuous scale, with both leukocyte counts and serum lipids log transformed (base e) to achieve a normal distribution in residuals. Because leukocyte counts were log transformed in ANCOVA analyses, results were reported as geometric means. Since both serum lipids and leukocyte counts were log transformed in multiple regression analyses, the slope was not easily interpretable and was thus also presented as a percent change in blood leukocyte count for a 10% increase in serum lipid level using the following equation:

% change in blood leukocyte count = (1.1^β^ − 1) × 100

where β is the slope from the regression model relating serum lipids to blood leukocytes. The use of 10% does not affect the significance of the results; rather, it was chosen to demonstrate the influence on blood leukocytes from a realistic and modest change in serum lipids. Analyses were adjusted for age, serum cotinine, BMI, waist circumference, race/ethnicity, education, statin use, and survey cycle, as these factors may influence lipid and leukocyte profiles [7,44,45,46,47,48,49]. A *p*-value less than 0.05 was considered statistically significant.

## 3. Results

### 3.1. Descriptive Statistics

Characteristics of the study population according to gender are presented in Table 1. The study population included a greater proportion of women than men (52.5% vs. 47.5%). Median age and the distribution of ethnicity groups were relatively similar between men and women. Median BMI additionally fell within the overweight range for both genders [50]. Serum cotinine levels, a marker of cigarette smoking [41], was higher and much more variable in men as compared to women, and a slightly greater percentage of men reported taking statins (11.7% vs. 9.0%). Accordingly, a greater percentage of men had total cholesterol levels <200 mg/dL (54.1% vs. 48.4%) and HDL-C within the optimal range (73.0% vs. 65%) as compared to women. Through analysis of differential leukocyte counts, women had a higher median level of lymphocytes and neutrophils, whereas men had higher median levels of monocytes and eosinophils. Median and interquartile range (IQR) values for each cell subset fell within standard reference ranges of blood leukocytes for adult men and women [51].

### 3.2. Associations between Serum Lipids and Leukocyte Counts in Men

To assess the relationship between serum lipids and leukocyte counts, data was analyzed by blood leukocyte subclass and clinically-relevant cutoffs for serum lipids [35,43]. In men, lymphocyte, monocyte, neutrophil, eosinophil, and basophil counts did not differ by levels of total cholesterol or LDL-C (Table 2). Conversely, men with low HDL-C (<40 mg/dL) had higher lymphocyte, neutrophil, eosinophil, and basophil counts, whereas men with elevated triglyceride levels had higher lymphocyte counts. Associations between triglyceride levels and neutrophil and basophil counts were additionally observed, as men with borderline high triglycerides (150 to <200 mg/dL) tended to have lower counts of these leukocyte subsets.

### 3.3. Associations between Serum Lipids and Leukocyte Counts in Women

When assessing the relationship between clinical lipid markers and leukocyte subset counts in women, greater overall associations were observed as compared to men, with the exception of HDL-C (Table 3). In women, increasing levels of total cholesterol, LDL-C, and triglycerides were associated with sequentially higher lymphocyte counts. Conversely, increasing LDL-C was associated with reduced monocyte and neutrophil counts—a somewhat unexpected observation given the independent, positive associations of LDL-C, monocytes, and neutrophils with CVD risk [52,53,54]. Significant associations between triglyceride levels and monocytes, neutrophils, and basophils were additionally observed, where elevated triglyceride levels corresponded to higher monocyte and basophil counts. Similar to what was observed in men, low HDL-C (<50 mg/dL) was associated with higher lymphocyte counts in women; however, associations between HDL-C and the other leukocyte subsets were not significant.

### 3.4. Total Cholesterol Predicts Leukocyte Counts in Women, But Not Men

Given the differential associations between blood lipids and leukocyte subsets between men and women, we next set out to determine whether blood lipids could be used to predict leukocyte counts as a way to further elucidate a potential direct relationship between lipid metabolism and immunity. Using both adjusted and unadjusted multiple regression models, total cholesterol (Table 4) levels were not able to significantly predict any of the leukocyte subset counts in men. Conversely, total cholesterol significantly predicted lymphocyte counts in women (Table 4), an observation that was strengthened after adjusting for age, serum cotinine, BMI, waist circumference, race/ethnicity, education, statin use, and NHANES survey cycle. In the adjusted model, a 1.6% increase in lymphocyte counts (10^3^ cells/μL) could be predicted for every 10% increase in total cholesterol level (mg/dL). Total cholesterol was not able to significantly predict monocytes, neutrophils, eosinophils, or basophils in women using the adjusted model.

### 3.5. LDL-Cholesterol Predicts Leukocyte Counts in Women, But Not Men

Similar to that observed for total cholesterol, LDL-C did not predict leukocyte counts in men using adjusted and unadjusted multiple regression models (Table 5). However, LDL-C served as a significant predictor of lymphocyte, monocyte, and neutrophil counts in women. In the adjusted model, every 10% increase in LDL-C was associated with a 0.6% increase in lymphocytes, as well as a 0.7% and 0.6% decrease in monocyte and neutrophil counts, respectively. These findings suggest that LDL-C may have a greater impact on global immune profiles, beyond total cholesterol. Predictive associations were not observed between LDL-C and eosinophil or basophil counts.

### 3.6. HDL-Cholesterol Predicts Leukocyte Counts in both Men and Women

Despite the greater associations between immune profiles and serum lipids observed in women described above, HDL-C served as a significant predictor of each leukocyte subset in both men and women in the unadjusted model (Table 6). In contrast to the positive associations observed between total cholesterol, LDL-C, and immune profiles, HDL-C was inversely associated with all predicted leukocyte levels. Interestingly, certain associations were lost after adjusting for covariates, which varied by gender. In the adjusted model, a 10% increase in HDL-C was associated with a 1.6% and 0.9% decrease in lymphocytes in men and women, respectively. However, predicted changes in neutrophils (−1.1%), eosinophils (−0.3%), and basophils (−0.1%) were only observed in men for every 10% increase in HDL-C, whereas predicted changes in monocytes (−0.6%) were solely observed in women. These findings correspond to the greater associations between HDL-C and leukocyte counts observed in men above (Table 2) and suggest that changes in HDL-C may have a greater magnitude and range of impact on immune profiles in men, as compared to women.

### 3.7. Serum Triglycerides Levels Predict Leukocyte Counts in Both Men and Women

In addition to blood cholesterol markers, triglycerides levels were found to predict leukocyte subsets through positive associations, with relatively minimal variability observed between men and women (Table 7). In adjusted models, a 10% increase in triglyceride levels was associated with a 1.1% and 1.4% increase in lymphocytes in men and women, respectively. Similar trends were observed for neutrophils and basophils, where a 10% increase in triglycerides corresponded to a predicted 0.7% and 0.1% increase in these cell subsets in men, respectively, as well as a 1.2% and 0.1% increase in women. Predicted changes in monocytes (0.6%) were only observed in women, whereas triglycerides were not able to predict changes in eosinophils in men or women.

## 4. Discussion

Clinical and mechanistic studies have demonstrated that lipid metabolism plays an important role in leukocyte activity, which may have significant gender-specific implications for CVD, metabolic dysfunction, and immune disorders [17,19,26,29,38,40,55]. In this study of 5647 men and women from NHANES 1999–2004, we have demonstrated that there are significant relationships between clinical lipid markers and leukocyte counts in the general U.S. population, and that clinical lipid markers differentially predict specific leukocyte subsets to varying degrees in men and women. Our findings suggest that hypertriglyceridemia may impact immune profiles in both men and women, whereas hypercholesterolemia may only affect lymphocyte profiles in women. Further, increases in HDL-C may differentially affect immune profiles in men and women, leading to variable immune outcomes. To our knowledge, our study is the first to evaluate the lipid–leukocyte relationship in NHANES.

Differences in both lipid and leukocyte profiles between men and women have previously been reported [56,57], suggesting that the gender-specific lipid-immune relationships we observed may be attributable to innate variability in lipid metabolism and immune cell behavior. Clinical guidelines for optimal HDL-C levels are distinct for men (>40 mg/dL) and women (>50 mg/dL) [35,36], and men often present with higher total cholesterol, LDL-C, and triglycerides as compared to premenopausal women, although these trends are equalized or reversed in post-menopausal women [56,58,59]. Gender-specific patterns in lipid profiles are in line with observed differences in CVD risk for men vs. women at different life stages, and some studies suggest that there are gender differences in the efficacy of statin therapy [37,60,61]. Women also have greater risk for autoimmune disorders, viral infection, and adverse responses to vaccines, while exhibiting enhanced humoral and cell-mediated responses as compared to men [38,39,40,62]. Variability in lipid and immune profiles between men and women has not been solely attributed to hormonal differences, but may be further impacted by differences in body fat distribution and cholesterol, triglyceride, and fatty acid metabolism [58,63,64,65]. Thus, the complex dynamics of lipid metabolism and immune function between genders may explain the variability in relationships between specific clinical biomarkers.

Lipoproteins can directly impact leukocyte profiles and activity through modulation of cellular cholesterol flux, lipid raft organization, sequestration of pathogenic stimuli, and provision of immunomodulatory proteins [19,22]. Cellular cholesterol loading—either through enhanced LDL uptake or deletion of HDL-associated lipid efflux transporters such as ABCA1 or ABCG1—has been shown to enhance the proliferative and inflammatory potential of lymphocytes and macrophages [24,26,66]. However, mechanisms of lipid metabolism and expression profiles differ in monocytes [67], and hypercholesterolemia has been shown to impair neutrophil function [68]. Similar trends were observed on our study, as increased total cholesterol and LDL-C levels were associated with greater predicted levels of lymphocytes in women. Conversely, greater LDL-C was associated with lower predicted monocyte and neutrophil levels in women, whereas no associations between total cholesterol, LDL-C, or any leukocyte subset were observed in men. Thus, hypercholesterolemia—particularly due to elevated LDL levels—may promote selective, and gender-specific expansion lymphocytes and altered, and potentially impaired, monocyte and neutrophil profiles. These observations may be due to gender-specific variability in lipid metabolism and immune responsiveness, as described above [56,57,64].

In contrast to cholesterol loading, HDL-mediated efflux has been shown to promote cholesterol depletion of leukocytes, leading to reduced formation of membrane lipid rafts, suppressed toll-like receptor 4-mediated inflammatory signaling, and a lower proliferative capacity of lymphocytes [24,25,26,69]. Further, lipoproteins—particularly HDL—can sequester bacterial pathogens such as lipopolysaccharide and lipoteichoic acid, thereby preventing leukocyte activation and improving clinical outcomes of sepsis [19,70,71,72,73,74]. HDL and apolipoprotein A 1 (apoA1) have additionally been shown to inhibit neutrophil activation, recruitment, and adhesion [75]. Our study revealed similar associations, in that a 10% increase in HDL-C predicted lower lymphocyte, neutrophil, eosinophil, and basophil counts in men. In contrast, after adjusting for potential confounding factors such as age, smoking, BMI, central adiposity (waist circumference), race/ethnicity, and statin use [7,44,45,46,47,48,49], HDL-C levels were only able to predict lymphocyte and monocyte counts in women. Regardless of gender, each lipid marker was inversely associated with HDL-C levels, suggesting that cholesterol- and immune-modulating properties of HDL universally suppress indices of leukocytosis in monocytes and lymphocyte subsets, and that HDL appears to have limited impacts on granulocyte populations in women. Interestingly, differences in HDL particle size profiles and cholesterol efflux capacity have been observed between genders, with women exhibiting a greater degree of larger HDL_2_- and scavenger receptor class B type I (SR-BI)-mediated cholesterol efflux, whereas men exhibit greater preβ HDL- and ATP-binding cassette transporter A1 (ABCA1)-mediated efflux [76]. Thus, the variable relationships between HDL-C and differential cell subsets may represent differences in HDL particle size profiles and leukocyte cholesterol efflux patterns between men and women.

In addition to observing unique relationships between serum cholesterol and immune markers between men and women, we found significant linear trends between triglycerides vs. lymphocytes, neutrophils, and basophils in both men and women, whereas positive linear trends between triglycerides vs. monocytes were only observed in women. Accordingly, a 10% increase in triglycerides was associated with higher predicted lymphocyte, neutrophil, and basophil counts in both men and women, as well as greater predicted monocyte counts in women. Elevated serum triglycerides are indicative of elevated hepatic lipogenesis, very low-density lipoprotein (VLDL) production, and reduced systemic lipolysis [77,78]. Hypertriglyceridemia is often associated with physiological conditions that promote immune activation, including metabolic dysfunction, lipid deposition in metabolic and lymphoid tissues, and direct activation of leukocyte populations by fatty acids [17,79,80,81]. Given the universal positive associations between serum triglycerides and leukocyte subsets, with little variability between men and women, our findings suggest that hypertriglyceridemia may promote leukocytosis in a relatively gender-independent manner.

Our study is one of a limited number of reports that has evaluated the direct, predictive relationship between lipid markers and leukocyte profiles in large-scale populations—particularly in the general U.S. population. In hypertensive male and female patients from the China Stroke Primary Prevention Trial cohort, Liu et al. [32] found that serum total cholesterol, LDL-C, and triglyceride levels were positively associated with total leukocyte, lymphocyte, and neutrophil counts, whereas HDL-C was inversely associated with leukocyte counts. In 3282 Taiwanese subjects participating in a standard hospital health screening program, hyperlipidemia was positively associated with total leukocyte counts, as well as each differential cell counts aside from eosinophils [33]. In evaluating immune associations by lipid category, hypertriglyceridemia had a stronger positive association with levels of each differential leukocyte subset. Conversely, hypercholesterolemia was positively associated with lymphocyte, neutrophil, and basophil counts, yet inversely associated with monocyte counts [33].

Together, these population studies support findings from our study and mechanistic trials, in that lipid profiles indicative of lipid loading promote selective leukocytosis, whereas HDL-mediated pathways are associated with suppressed leukocytosis [19,24,25,69]. However, the majority of previous studies have not elucidated gender-specific relationships between markers of immunity and serum lipids [32,33], which we found to be significantly variable. In line with our study, Oda et al. [34] observed greater associations between hypertriglyceridemia, hypo-HDL cholesterolemia, and hyper-LDL cholesterolemia vs. differential leukocyte counts in Japanese men participating in general health screenings, as compared to women. While we observed stronger overall lipid-leukocyte associations in women—with the exception of HDL-C—this report supports our conclusions that complex, gender-specific associations between serum lipid markers and leukocyte counts exist at the population level.

## 5. Conclusions

The predictive, diagnostic, and therapeutic significance of our findings warrants further investigation. Global and/or selective elevation of leukocyte populations may be indicative of CVD [6], metabolic dysfunction [9,10], infection [2], injury [3], or allergy [5]. While a limitation of our study was that we were unable to account for confounding immune-modulating factors (e.g., infection and allergy status, steroid usage), we clearly demonstrate that serum lipid levels impact and predict leukocyte profiles, suggesting that lipid-modulating therapies—such as diet, weight loss, or statins—may regulate leukocyte expansion and behavior [17,19,55,82,83,84]. Importantly, the efficacy of targeting serum lipids to promote immune health may be gender specific, calling for greater examination of immune outcomes and gender effects in clinical lipid trials.

## Figures and Tables

**Table 1:** Descriptive statistics of men and women in NHANES 1999–2004 (*n* = 5647).

		Men			Women			*p*-Value
			IQR			IQR		
		Median	Lower	Upper	Median	Lower	Upper	
Subjects (*n* (%))		2682	(47.5)		2965	(52.5)		
Age, years		43	32	56	44	33	58	0.0005
Race/ethnicity (*n* (%))								0.0152
	Hispanic	739	(27.6)		821	(27.7)		
	Non-Hispanic White	1378	(51.4)		1499	(50.6)		
	Non-Hispanic Black	470	(17.5)		542	(18.3)		
	Other	95	(3.5)		103	(3.5)		
BMI (kg/m^2^)		27.1	24.2	30.3	26.6	23.1	31.5	0.2536
Waist circumference (cm)		97.8	89.4	107.6	90.9	81.1	102.3	<0.0001
Serum cotinine (ng/mL)		0.20	0.03	119.19	0.06	0.03	1.39	<0.0001
Statin use (*n* (%))								0.0049
	No	2368	(88.3)		2698	(91.0)		
	Yes	314	(11.7)		267	(9.0)		
Fasting serum lipids (mg/dL)								
	Total cholesterol	195.6	171.7	220.6	197.6	174.0	225.7	0.0066
	LDL-cholesterol	120.5	97.7	144.0	115.2	93.9	139.9	0.0007
	HDL-cholesterol	44.6	38.3	52.8	55.1	45.1	66.6	<0.0001
	Triglycerides	120.7	84.4	173.7	108.4	76.6	157.6	<0.0001
Total cholesterol (*n* (%))								0.1876
	<200 mg/dL	1451	(54.1)		1436	(48.4)		
	200 to <240 mg/dL	880	(32.8)		978	(33.0)		
	>240 mg/dL	351	(13.1)		551	(18.6)		
LDL-cholesterol (*n* (%))								0.0058
	<100 mg/dL	727	(27.1)		862	(29.1)		
	100 to <130 mg/dL	905	(33.7)		1032	(34.8)		
	130 to <160 mg/dL	683	(25.5)		684	(23.1)		
	160 to <190 mg/dL	274	(10.2)		268	(9.0)		
	>190 mg/dL	93	(3.5)		119	(4.0)		
HDL-cholesterol (*n* (%))								<0.0001
	M: ≥40 mg/dL; W: ≥50 mg/dL	1959	(73.0)		1926	(65.0)		
	M: <40 mg/dL; W: <50 mg/dL	723	(27.0)		1039	(35.0)		
Triglycerides (*n* (%))								0.074
	<150 mg/dL	1763	(65.7)		1966	(66.3)		
	150 to <200 mg/dL	434	(16.2)		495	(16.7)		
	≥200 mg/dL	485	(18.1)		504	(17.0)		
Blood leukocyte counts (10^3^ cells/μL)								
Lymphocytes		1.76	1.43	2.18	1.81	1.48	2.27	0.0003
Monocytes		0.50	0.40	0.62	0.44	0.34	0.55	<0.0001
Neutrophils		3.65	2.91	4.61	3.81	2.96	4.87	0.0064
Eosinophil		0.13	0.06	0.22	0.10	0.04	0.18	<0.0001
Basophil		0.00	0.00	0.01	0.00	0.00	0.03	0.0025

BMI: body mass index; IQR: interquartile range; M: men; NHANES: National Health and Nutrition Examination Survey; W: women. *p*-values were generated from Chi-square tests and *t*-tests for categorical and continuous variables, respectively.

**Table 2:** Serum lipids are differentially associated with peripheral leukocyte counts in men from NHANES 1999–2004.

	Lymphocytes			Monocytes			Neutrophils			Eosinophils			Basophils		
	GM	95% CI		GM	95% CI		GM	95% CI		GM	95% CI		GM	95% CI	
Total cholesterol (mg/dL)															
<200	1.85	1.79	1.91	0.55	0.53	0.57	3.66	3.55	3.78	1.22	1.20	1.25	1.03	1.02	1.03
200 to <240	1.84	1.78	1.90	0.54	0.52	0.56	3.79	3.63	3.96	1.23	1.20	1.26	1.03	1.02	1.04
>240	1.93	1.84	2.03	0.54	0.51	0.56	3.81	3.62	4.00	1.23	1.21	1.25	1.03	1.03	1.04
*p*-trend	0.17			0.13			0.72			0.20			0.92		
LDL-cholesterol (mg/dL)															
<100	1.86	1.80	1.93	0.56	0.53	0.58	3.78	3.64	3.92	1.23	1.21	1.25	1.03	1.03	1.04
100 to <130	1.83	1.76	1.90	0.53	0.52	0.55	3.64	3.49	3.78	1.23	1.21	1.25	1.03	1.02	1.03
130 to <160	1.85	1.78	1.93	0.54	0.52	0.57	3.73	3.59	3.88	1.22	1.19	1.25	1.03	1.02	1.03
160 to <190	1.88	1.80	1.96	0.53	0.51	0.56	3.72	3.52	3.93	1.23	1.21	1.26	1.03	1.02	1.04
>190	1.98	1.80	2.17	0.55	0.50	0.62	3.79	3.36	4.27	1.21	1.17	1.26	1.03	1.02	1.05
*p*-trend	0.33			0.24			0.96			0.50			0.89		
HDL-cholesterol (mg/dL)															
≥40	1.82	1.76	1.88	0.54	0.52	0.56	3.67	3.54	3.79	1.22	1.20	1.24	1.03	1.02	1.03
<40	1.95	1.89	2.01	0.55	0.52	0.57	3.83	3.70	3.98	1.24	1.22	1.26	1.03	1.03	1.04
*p*-value	0.0004			0.60			0.0114			0.05			0.0077		
Triglyceride (mg/dL)															
<150	1.80	1.75	1.84	0.55	0.53	0.57	3.74	3.61	3.86	1.23	1.21	1.25	1.03	1.03	1.03
150 to <200	1.93	1.85	2.01	0.56	0.53	0.58	3.66	3.53	3.78	1.22	1.20	1.25	1.03	1.02	1.03
≥200	1.97	1.89	2.05	0.53	0.51	0.56	3.74	3.50	4.01	1.22	1.19	1.24	1.03	1.02	1.04
*p*-trend	< 0.0001			0.26			0.05			0.41			0.035		

CI: confidence interval; GM: geometric mean; NHANES: National Health and Nutrition Examination Survey. Clinical ranges for blood lipids levels are presented as mg/dL, and clinical blood leukocyte subsets are presented as geometric means (10^3^ cells/μL). Geometric means and tests for linear trend were adjusted for age, serum cotinine, BMI, waist circumference, race/ethnicity, education, statin use, and survey cycle.

**Table 3:** Serum lipids are differentially associated with peripheral leukocyte counts in women from NHANES 1999–2004.

	Lymphocytes			Monocytes			Neutrophils			Eosinophils			Basophils		
	GM	95% CI		GM	95% CI		GM	95% CI		GM	95% CI		GM	95% CI	
Total cholesterol (mg/dL)															
<200	1.94	1.87	2.01	0.50	0.49	0.51	3.77	3.65	3.89	1.19	1.17	1.20	1.03	1.02	1.03
200 to <240	2.00	1.94	2.07	0.48	0.46	0.50	4.11	3.93	4.30	1.18	1.16	1.20	1.03	1.03	1.04
>240	2.08	1.20	2.17	0.49	0.46	0.52	4.21	3.97	4.46	1.20	1.18	1.22	1.04	1.04	1.05
*p*-trend	0.0008			0.14			0.35			0.75			0.18		
LDL-cholesterol (mg/dL)															
<100	1.93	1.86	1.21	0.51	0.49	0.52	4.01	3.84	4.18	1.19	1.18	1.20	1.03	1.03	1.04
100 to <130	1.99	1.92	2.06	0.49	0.47	0.51	3.90	3.75	4.05	1.18	1.17	1.20	1.03	1.03	1.04
130 to <160	2.00	1.92	2.09	0.48	0.46	0.50	3.83	3.64	4.04	1.19	1.17	1.20	1.03	1.02	1.04
160 to <190	2.06	1.93	2.20	0.49	0.46	0.52	3.86	3.56	4.18	1.17	1.15	1.19	1.04	1.03	1.04
>190	2.09	1.95	2.23	0.46	0.42	0.52	3.71	3.40	4.06	1.22	1.15	1.28	1.04	1.02	1.05
*p*-trend	0.0045			0.0089			0.046			0.92			0.51		
HDL-cholesterol (mg/dL)															
≥50	1.93	1.88	1.99	0.49	0.47	0.51	3.89	3.75	4.03	1.18	1.17	1.20	1.03	1.03	1.04
<50	2.03	1.95	2.12	0.50	0.41	0.51	3.97	3.81	4.15	1.20	1.18	1.21	1.03	1.03	1.04
*p*-trend	0.0079			0.36			0.25			0.08			0.29		
Triglycerides (mg/dL)															
<150	1.88	1.82	1.93	0.48	0.46	0.50	3.97	3.84	4.10	1.19	1.18	1.20	1.03	1.03	1.04
150 to <200	2.06	1.98	2.14	0.50	0.48	0.52	3.84	3.66	4.02	1.18	1.17	1.20	1.03	1.03	1.04
≥200	2.18	2.06	2.30	0.53	0.51	0.56	3.91	3.67	4.18	1.19	1.16	1.21	1.04	1.03	1.04
*p*-trend	<0.0001			<0.0001			<0.0001			0.41			0.0002		

CI: confidence interval; GM: geometric mean; NHANES: National Health and Nutrition Examination Survey. Clinical ranges for blood lipids levels are presented as mg/dL, and clinical blood leukocyte subsets are presented as geometric means (10^3^ cells/μL). Geometric means and tests for linear trend were adjusted for age, serum cotinine, BMI, waist circumference, race/ethnicity, education, statin use, and survey cycle.

**Table 4:** Multiple regression models of serum total cholesterol levels predicting changes in peripheral blood leukocyte counts between men and women in NHANES 1999–2004.

		Men				Women			
Cell Count	Model	β	SE	% Δ	*p*	β	SE	% Δ	*p*
Lymphocytes	unadjusted	0.045	0.046	0.4	0.33	0.083	0.035	0.8	<0.05
	adjusted	0.081	0.044	0.8	0.07	0.168	0.036	1.6	<0.0001
Monocytes	unadjusted	−0.038	0.037	−0.4	0.30	0.002	0.037	0.0	0.95
	adjusted	−0.064	0.036	−0.6	0.08	−0.079	0.040	−0.7	0.06
Neutrophils	unadjusted	0.051	0.045	0.5	0.27	−0.038	0.045	−0.4	0.40
	adjusted	0.002	0.042	0.0	0.97	−0.035	0.047	−0.3	0.46
Eosinophils	unadjusted	−0.012	0.017	−0.1	0.49	−0.006	0.015	−0.1	0.67
	adjusted	−0.014	0.017	−0.1	0.41	−0.012	0.017	−0.1	0.49
Basophils	unadjusted	0.009	0.006	0.1	0.18	0.013	0.006	0.1	<0.05
	adjusted	0.006	0.006	0.1	0.34	0.006	0.005	0.1	0.27

β: slope; SE: standard error; *p*: *p*-value. % Δ = the predicted % change in blood leukocyte count (10^3^ cells/μL) for a 10% increase in total cholesterol level (mg/dL). Adjusted model is adjusted for age, serum cotinine, BMI, waist circumference, race/ethnicity, education, statin use, and survey cycle. To best approximate linearity, both predictor and outcome were log transformed (base e); thus slope and standard errors are on log scale.

**Table 5:** Multiple regression models of serum LDL-C predicting changes in peripheral blood leukocyte counts between men and women in NHANES 1999–2004.

		Men				Women			
Cell Count	Model	β	SE	% Δ	*p*	β	SE	% Δ	*p*
Lymphocytes	unadjusted	0.025	0.032	0.2	0.43	0.063	0.026	0.6	<0.05
	adjusted	0.037	0.031	0.3	0.24	0.063	0.026	0.6	<0.05
Monocytes	unadjusted	−0.019	0.026	−0.2	0.47	−0.023	0.023	−0.2	0.33
	adjusted	−0.035	0.026	−0.3	0.19	−0.075	0.023	−0.7	<0.01
Neutrophils	unadjusted	0.017	0.031	0.2	0.59	−0.036	0.030	−0.3	0.25
	adjusted	−0.008	0.029	−0.1	0.80	−0.065	0.031	−0.6	<0.05
Eosinophils	unadjusted	−0.007	0.011	−0.1	0.50	−0.002	0.010	0.0	0.85
	adjusted	−0.006	0.011	−0.1	0.61	−0.007	0.012	−0.1	0.56
Basophils	unadjusted	0.004	0.004	0.0	0.35	0.004	0.003	0.0	0.28
	adjusted	0.003	0.004	0.0	0.48	−0.002	0.003	0.0	0.56

β: slope; SE: standard error; *p*: *p*-value. % Δ = the predicted % change in blood leukocyte count (10^3^ cells/μL) for a 10% increase in LDL-C level (mg/dL). Adjusted model is adjusted for age, serum cotinine, BMI, waist circumference, race/ethnicity, education, statin use, and survey cycle. To best approximate linearity, both predictor and outcome were log transformed (base e); thus slope and standard errors are on log scale.

**Table 6:** Multiple regression models of serum HDL-cholesterol predicting changes in peripheral blood leukocyte counts between men and women in NHANES 1999–2004.

		Men				Women			
Cell Count	Model	β	SE	% Δ	*p*	β	SE	% Δ	*p*
Lymphocytes	unadjusted	−0.204	0.035	−1.9	<0.0001	−0.234	0.027	−2.2	<0.0001
	adjusted	−0.171	0.032	−1.6	<0.0001	−0.098	0.033	−0.9	<0.01
Monocytes	unadjusted	−0.067	0.031	−0.6	<0.05	−0.101	0.021	−1.0	<0.0001
	adjusted	−0.018	0.033	−0.2	0.59	−0.059	0.022	−0.6	<0.01
Neutrophils	unadjusted	−0.192	0.032	−1.8	<0.0001	−0.205	0.031	−1.9	<0.0001
	adjusted	−0.118	0.031	−1.1	<0.001	−0.049	0.035	−0.5	0.17
Eosinophils	unadjusted	−0.035	0.012	−0.3	<0.01	−0.029	0.009	−0.3	<0.01
	adjusted	−0.032	0.012	−0.3	<0.05	−0.019	0.010	−0.2	0.06
Basophils	unadjusted	−0.014	0.003	−0.1	<0.001	−0.009	0.004	−0.1	<0.05
	adjusted	−0.011	0.004	−0.1	<0.01	−0.002	0.004	0.0	0.64

β: slope; SE: standard error; *p*: *p*-value. % Δ = the predicted % change in blood leukocyte count (10^3^ cells/μL) for a 10% increase in LDL-C level (mg/dL). Adjusted model is adjusted for age, serum cotinine, BMI, waist circumference, race/ethnicity, education, statin use, and survey cycle. To best approximate linearity, both predictor and outcome were log transformed (base e); thus slope and standard errors are on log scale.

**Table 7:** Multiple regression models of serum triglycerides predicting changes in peripheral blood leukocyte counts between men and women in NHANES 1999–2004.

		Men				Women			
Cell Count	Model	β	SE	% Δ	*p*	β	SE	% Δ	*p*
Lymphocytes	unadjusted	0.097	0.012	0.9	<0.0001	0.135	0.014	1.3	<0.0001
	adjusted	0.112	0.013	1.1	<0.0001	0.145	0.015	1.4	<0.0001
Monocytes	unadjusted	0.025	0.013	0.2	0.06	0.100	0.017	1.0	<0.0001
	adjusted	0.000	0.014	0.0	1.00	0.067	0.019	0.6	<0.001
Neutrophils	unadjusted	0.123	0.018	1.2	<0.0001	0.161	0.017	1.6	<0.0001
	adjusted	0.075	0.020	0.7	<0.001	0.124	0.020	1.2	<0.0001
Eosinophils	unadjusted	0.016	0.006	0.2	<0.05	0.012	0.005	0.1	<0.05
	adjusted	0.010	0.006	0.1	0.10	0.006	0.005	0.1	0.21
Basophils	unadjusted	0.010	0.002	0.1	<0.0001	0.013	0.002	0.1	<0.0001
	adjusted	0.008	0.002	0.1	<0.01	0.011	0.002	0.1	<0.0001

β: slope; SE: standard error; *p*: *p*-value. % Δ = the predicted % change in blood leukocyte count (10^3^ cells/μL) for a 10% increase in LDL-C level (mg/dL). Adjusted model is adjusted for age, serum cotinine, BMI, waist circumference, race/ethnicity, education, statin use, and survey cycle. To best approximate linearity, both predictor and outcome were log transformed (base e); thus slope and standard errors are on log scale.
